# Nutritional status coverage trend registered in the SISVAN web in seven municipalities of the Zona Da Mata Mineira, Brazil, from 2008 to 2017, and its association with socio-economic, demographic and organisation of health system variables

**DOI:** 10.1017/jns.2019.42

**Published:** 2020-01-20

**Authors:** Irene da Silva Araújo Gonçalves, Patrícia Feliciano Pereira, Mariana Belcavelo Lino Silva, Fernanda Batista Ladeira, Tiago Ricardo Moreira, Rosângela Minardi Mitre Cotta, Glauce Dias da Costa

**Affiliations:** 1 Departamento de Nutrição e Saúde, Universidade Federal de Viçosa, UFV. Av. P.H. Rolfs, s/n, CCB II, DNS, Centro, Viçosa-MG, 36570-900, Brazil; 2 Departamento de Medicina e Enfermagem, Universidade Federal de Viçosa, UFV. Av. P.H. Rolfs, s/n, DEM, Centro, Viçosa-MG, 36570-900, Brazil

**Keywords:** Nutrition policy, Nutritional surveillance, Coverage of public health services, Public health, ACS, *agentes comunitários de saúde* (community health agents), DATASUS, Departamento de Informática do SUS (Department of Informatics of SUS), e-SUS AB, e-SUS Primary Care, ESF, Estratégia Saúde da Família (Family Health Strategy), GDP, gross domestic product, HDI-M, Municipal Human Development Index, NASF, Extended Core of Family Health, NASF-AB, Núcleo Ampliado de Saúde da Família e Atenção Básica (Expanded Core of Family Health and Primary Care), PBF, Programa Bolsa Família (Bolsa Family Programme), SISVAN, Sistema Nacional de Vigilância Alimentar e Nutricional (Food and Nutrition Surveillance System), SUS, Sistema Único de Saúde (Unified Health System), VAN, Vigilância Alimentar e Nutricional (Food and Nutrition Surveillance)

## Abstract

We analysed the coverage trend of the evaluation of the nutritional status of users of public health services registered in the Food and Nutrition Surveillance System (SISVAN) between 2008 and 2017 in seven municipalities and verified the association of the coverage trend with the socio-economic, demographic and organisational aspects of health system variables. It is an ecological time-series study performed with secondary data extracted from health information systems. Descriptive statistics, linear regression model and repeated measures analysis were performed. The coverage of evaluation of nutritional status was low over the period. Five municipalities showed a tendency to increase coverage, although small, while two remained stable. The highest annual variation in coverage increase was concentrated in the group of pregnant women and the lowest in adolescents and older adults. There was a downward trend in follow-ups from the Bolsa Family Programme and a trend towards increased follow-ups from SUS Primary Care (e-SUS AB). SISVAN coverage was positively associated with the proportion of rural population (*P* ≤ 0·001) and coverage of community health agents (*P* < 0·001); and negatively associated with total population (*P* < 0·001), demographic density (*P* = 0·006) and gross domestic product per capita (*P* = 0·008). Despite the tendency to increase coverage in some municipalities, SISVAN still presents low coverage of nutritional status assessment, which compromises population monitoring. Knowing the factors that influence the coverage can subsidise the elaboration of strategies for its expansion.

The epidemiological and demographic transition experienced in recent years^(1)^ has resulted in an unclear and complex aspect of food and nutritional insecurity. A reduction in malnutrition coexists with a significant increase in the prevalence of an overweight population^(2)^.

Population surveys have shown a tendency of reduced malnutrition, associated with an increase in obesity, as well as the coexistence of the two scenarios at different stages of life, especially in childhood^(3–6)^. This health situation is marked by an accelerated demographic transition. Further, this trend is associated with an epidemiological transition characterised by unresolved infectious disease, malnutrition, reproductive health problems, and the hegemonic presence of chronic non-communicable diseases and their risk factors (including inadequate nutrition, smoking, obesity and physical inactivity)^(7)^.

A food and nutrition surveillance system is essential for detecting health problems related to food and nutrition and for monitoring the implementation of public policies for both development and emergency programmes^(4)^. However, despite regional efforts by the WHO since 1980 to encourage member states to develop food and nutrition surveillance systems, only a few countries have responded positively and incorporated these systems; these implementations are still in a primary stage, resulting in important gaps in relation to the data collection to evaluate the nutritional state of the population^(8,9)^.

According to the Global Nutrition Report (2017)^(10)^ the average follow-up of nutritional status in different countries is not enough to identify vulnerable groups and enable the construction of the dialogues, partnerships, actions and responsibilities needed to end the nutritional problems and solve the health issues of the population.

In Brazil, the population and geographical coverage of the Food and Nutrition Surveillance System (Sistema Nacional de Vigilância Alimentar e Nutricional; SISVAN) is still low^(11–13)^. Research so far has identified only the use of the system in order to receive resources for care programmes^(14)^, as well as the underutilisation of SISVAN information within the scope of the Basic Management of Basic Health, as identified by the Unified Health System (Sistema Único de Saúde; SUS)^(15)^.

Thus, the present study analysed the coverage trend of the evaluation of the nutritional status of users of public health services registered in the SISVAN web between 2008 and 2017 in seven municipalities of the Zona da Mata, Minas Gerais, and verified the association of the coverage trend with the socio-economic, demographic and organisational aspects of health system variables.

## Methods

This ecological time-series study assessed the coverage of the nutritional status recorded in the SISVAN web in seven municipalities in the Zona da Mata, Minas Gerais (Manhuaçu, Cataguases, Muriaé, Ponte Nova, Viçosa, Juiz de Fora, and Ubá) over 10 years (2008 to 2017). The population comprised 1 087 953 inhabitants distributed in a territorial area of 4576·84/km^2^, with about 7·55 % residing in a rural area. The municipalities were selected because they are centres of health of microregions of Zona da Mata and consistently represent the population of this region.

The data sources were as follows: the SISVAN web virtual site (https://aps.saude.gov.br/ape/vigilanciaalimentar/sisvan/); the SUS Department of Information Technology (Departamento de Informática do SUS; DATASUS) (http://datasus.saude.gov.br/datasus); the Brazilian Institute of Geography and Statistics (https://cidades.ibge.gov.br/); the Department of Primary Health Care of the Ministry of Health (http://dab.saude.gov.br/portaldab/); the National Registry of Health Establishments (http://cnes.datasus.gov.br/); the Atlas of Human Development in Brazil, produced by the United Nations Development Programme (http://atlasbrasil.org.br/2013/); and the Live Birth Information System (Sistema de Informações sobre Nascidos Vivos; SINASC; http://www2.datasus.gov.br/DATASUS/index.php?area=060702). These data sources are in the public domain and were collected from May to June 2018.

The indicators constructed to assess the performance of the system in relation to the monitoring of nutritional status were life-stage coverage, total coverage, proportion of nutritional follow-up from Bolsa Family Programme (Programa Bolsa Família; PBF) data, and proportion of data participation coming from e-SUS Primary Care (e-SUS AB). Total coverage refers to the percentage of individuals monitored in the SISVAN web and was obtained by dividing the number of individuals registered with nutritional status in the SISVAN web during the study period by the total resident population in the same period and multiplying it by 100. Life-stage coverage encompassed the following groups: preschool children (0–5 years), school-aged children (5–9 years), adolescents (10–19 years), adults (20–59 years), older adults (60 years or over), and pregnant women. For all groups but that of pregnant women, coverage was calculated by dividing the number of individuals in each age group registered with nutritional status in the SISVAN web during the study period by the total resident population of the same age group in the same period and multiplying by 100. Because of the lack of census and DATASUS data for the number of pregnant women, this estimate was calculated by the proposal of Nascimento^(15)^, which used the SINASC database. The number of live births from the previous year was used, plus 10 % (which referred to losses due to abortions and underreporting) and multiplied by the percentage of the female population of childbearing age (10–49 years). The proportion of follow-ups from the PBF and e-SUS AB was calculated by dividing the total number of nutritional status follow-ups from the PBF or e-SUS AB during the evaluation period by the total nutritional status follow-ups registered in the SISVAN web in the same period and multiplying by 100.

The sociodemographic and health variables used to evaluate the association with the total coverage of nutritional status monitoring were as follows: resident population, proportion of population living in rural areas, infant mortality rate, Municipal Human Development Index (HDI-M); gross domestic product (GDP); GDP per capita; immunisation coverage; resident population; demographic density; estimates of the population of the SUS covered by community health agents (*agentes comunitários de saúde*; ACS) and the Family Health Strategy (Estratégia Saúde da Família; ESF), the number of nutritionists who provide care by SUS, and the number of Expanded Core of Family Health and Primary Care (Núcleo Ampliado de Saúde da Família e Atenção Básica; NASF-AB) centres. These variables were categorised by the median in order to obtain two comparison groups.

The association was verified using variables from the year 2010, because the largest number of variables were available for that year. The NASF-AB number was relative only to the year 2017, because this nucleus had not yet been implemented in most municipalities in 2010.

The coverage and estimates of temporal variation were analysed by means of descriptive statistics (relative frequency) and a linear regression model, with total coverage being the outcome and the year being the explanatory variable. The CI was used to evaluate the statistical significance of temporal variations. The tendency of increase, decline or stagnation was expressed as annual coefficient of variation, with the respective 95 % CI. The trend with a regression coefficient not different from zero (*P* > 0·05) was considered stationary.

A repeated-measures ANOVA was conducted to calculate the influence of the explanatory variables (proportion of total population, total resident population, NASF-AB number, infant mortality rate, proportion of ACS coverage, proportion of ESF coverage, GDP per capita, immunisation coverage, and number of nutritionists in the SUS) on the dependent variable coverage of the monitoring of nutritional status, based on time, from 2008 to 2017. All data were analysed using Statistical Package for Social Sciences software 21.0, with a significance level of 5 %.

### Ethical standards disclosure

The present study is part of a larger project, entitled ‘Health Surveillance: Evaluation of Prevention Practices of Diseases and Health Promotion in the Zona da Mata Mineira’. The present study was conducted according to the guidelines laid down in the Declaration of Helsinki and all procedures involving research study participants were approved by the Ethics Committee of the Viçosa Federal University under the opinion 2 370 346 of 8 November 2017.

## Results

The socio-economic, demographic and health characteristics of the municipalities are presented in Table 1. Homogeneity was observed in the classification of the HDI-M, except in the municipality of Manhuaçu, which presented a medium HDI-M; the other municipalities were classified with a high HDI-M. The coverage by the ESF varied from 59·71 to 100 %, and the coverage by ACS ranged from 38·7 to 100 %. Low immunisation coverage and heterogeneity were observed in the population residing in rural areas between the municipalities.

**Table 1.** Characterisation of the municipalities that are health centres in Zona da Mata, Minas Gerais, according to socio-economic, demographic and organisational aspects of health system variables, 2017*

Municipality	Resident population (inhabitants)	Population density (inhabitants/km^2^)	HDI-M†	GDP per capita (R$)‡	Population covered by ACS (%)	Population covered by ESF (%)	No. of NASF-AB	Nutritionists in SUS (*n*)	Immunisation coverage (%)	Rural population (%)
Cataguases	75 025	155·0	0·751	17 337·3	70·8	92·8	2	10	42·5	4·3
Juiz de Fora	563 769	392·2	0·778	24 323·6	48·4	59·8	0	65	43·6	1·1
Manhuaçu	88 580	140·9	0·689	19 242·4	100	80·5	0	11	45·0	18·5
Muriaé	108 537	128·2	0·734	16 488·2	81·1	98·0	6	30	27·0	7·5
Ponte Nova	60 361	128·1	0·717	19 000·9	91·7	77·7	2	19	35·2	10·8
Ubá	113 300	276·8	0·724	21 342·1	38·7	59·7	2	19	43·9	3·8
Viçosa	78 381	261·3	0·775	15 604·4	62·7	84·7	1	19	47·0	6·8

HDI-M, Municipal Human Development Index; GDP, gross domestic product; ACS, *agentes comunitários de saúde* (community health agents); ESF, Estratégia Saúde da Família (Family Health Strategy); NASF-AB, Núcleo Ampliado de Saúde da Família e Atenção Básica (Expanded Core of Family Health and Primary Care); SUS, Sistema Único de Saúde (Unified Health System).

* Source of variables: Brazilian Institute of Geography and Statistics (Instituto Brasileiro de Geografia e Estatística; IBGE) (resident population, population density, GDP per capita); Atlas of Human Development (HDI-M); Department of Primary Care (population covered by ACS and ESF).

† Data for 2010.

† Data for 2013.

Table 2 presents the coverage of nutritional status monitoring in the different stages of life for each municipality, the mean annual variation, and the respective CI. All municipalities presented monitoring of nutritional status registered on the SISVAN web at all life stages during the study period.

**Table 2.** Temporal variation of the nutritional status monitoring coverage in the Food and Nutrition Surveillance System (Sistema Nacional de Vigilância Alimentar e Nutricional; SISVAN) web, according to life stage in the health centre municipalities of Zona da Mata, Minas Gerais, 2008–2017

Municipality and life stage	Coverage of monitoring of nutritional status (%)											
	2008	2009	2010	2011	2012	2013	2014	2015	2016	2017	Mean annual variation (%)*	95 % CI
Cataguases												
<5 years	7·9	20·6	20·0	25·8	28·0	51·3	40·97	60·7	NA	NA	6·8	4·2, 9·3
5–9 years	7·3	13·2	12·1	18·6	14·5	25·8	29·71	31·6	NA	NA	3·5	2·3, 4·6
10–19 years	6·0	10·0	8·1	12·0	8·6	22·0	18·55	17·0	NA	NA	1·9	0·5, 3·3
20–59 years	3·5	11·7	7·2	11·7	10·1	13·1	9·64	8·4	NA	NA	0·5	−0·7, 1·6
>60 years	0·0	14·5	3·4	12·0	8·7	6·3	0·8	0·7	NA	NA	−0·7	−2·8, 1·4
Pregnant women†	3·2	31·0	14·7	16·7	80·0	96·4	137·1	144·7	NA	NA	21·8	13·8, 29·8
Total coverage	4·0	12·6	8·1	13·2	11·5	17·1	13·4	13·6	14·6	19·0	1·2	0·4, 1·8
PBF contribution	99·8	39·6	73·5	48·2	49·9	64·2	85·6	73·2	64·3	42·6	−1·3	−6·5, 3·8
e-SUS AB contribution	NA	NA	NA	NA	NA	NA	NA	0·1	13·6	6·6	0·9	0·0, 1·9
Juiz de Fora												
<5 years	12·1	15·9	11·8	4·5	4·3	17·6	8·4	4·3	NA	NA	−0·9	−2·9, 1·1
5–9 years	11·4	10·2	8·1	5·6	5·8	7·5	6·8	7·5	NA	NA	−0·6	−1·2, 0·1
10–19 years	0·8	0·8	0·4	0·2	0·3	0·9	0·8	2·2	NA	NA	0·1	−0·1, 0·4
20–59 years	0·7	0·7	0·4	0·2	0·2	0·6	0·6	1·7	NA	NA	0·1	−0·1, 0·3
>60 years	0·8	1·3	0·4	0·4	0·2	2·7	0·7	0·4	NA	NA	0·0	−0·3, 0·3
Pregnant women†	10·5	16·5	8·8	4·2	7·0	27·0	21·6	16·6	NA	NA	1·5	−1·3, 4·3
Total coverage	2·3	2·5	1·7	0·9	0·9	2·6	1·7	2·2	4·73	4·99	0·3	−0·0, 0·6
PBF contribution	53·7	50·3	58·8	68·6	66·9	49·6	63·75	74·2	43·7	51·9	−0·1	−2·8, 2·6
e-SUS AB contribution	NA	NA	NA	NA	NA	NA	NA	12·5	50·4	46·6	5·1	1·5, 8·6
Manhuaçu												
<5 years	8·5	14·7	26·1	24·6	22·2	61·1	60·8	79·9	NA	NA	9·9	6·0, 13·9
5–9 years	9·2	10·0	17·5	16·1	13·0	22·2	25·6	28·6	NA	NA	2·7	1·5, 3·8
10–19 years	6·4	4·9	10·7	8·7	10·0	19·7	18·1	20·4	NA	NA	2·3	1·3, 3·3
20–59 years	4·1	5·3	11·8	9·2	9·0	11·4	10·3	13·8	NA	NA	1·1	0·3, 1·9
>60 years	0·6	0·6	6·9	2·5	9·7	26·7	19·9	36·1	NA	NA	4·9	2·6, 7·2
Pregnant women†	2·0	2·7	13·8	5·2	39·1	54·3	83·3	68·1	NA	NA	12·2	7·1, 17·2
Total coverage	5·1	6·1	13·1	10·5	11·2	20·2	19·1	24·7	27·9	40·0	3·4	2·4, 4·4
PBF contribution	89·2	76·7	57·9	81·9	78·4	63·0	64·1	44·8	35·2	19·4	−6·4	−9·4, −3·4
e-SUS AB contribution	NA	NA	NA	NA	NA	NA	NA	21·0	29·3	25·3	3·3	1·3, 5·3
Muriaé												
<5 years	11·9	14·9	18·7	29·1	27·8	55·0	68·8	71·8	NA	NA	9·5	6·5, 12·5
5–9 years	11·0	10·4	7·7	11·7	12·4	30·8	28·8	37·9	NA	NA	4·2	1·9, 6·5
10–19 years	7·0	7·4	5·6	6·4	6·3	31·3	19·3	21·5	NA	NA	2·8	0·1, 5·6
20–59 years	4·7	6·1	4·2	5·3	5·8	7·0	7·9	8·1	NA	NA	0·5	0·2, 0·8
>60 years	0·0	3·2	0·3	0·1	0·0	0·0	0·7	3·2	NA	NA	0·1	−0·5, 0·7
Pregnant women†	19·7	2·8	3·3	8·8	30·6	72·3	74·3	57·7	NA	NA	10·1	3·1, 17. 2
Total coverage	5·7	7·0	5·2	7·0	7·3	14·9	14·4	15·7	12·5	20·2	1·5	0·9, 2·2
PBF contribution	100·0	87·3	85·8	85·6	80·0	50·8	65·9	53·9	51·0	29·9	−6·9	−8·9, −4·8
e-SUS AB contribution	NA	NA	NA	NA	NA	NA	NA	24·6	27·3	9·1	2·4	0·2, 4·6
Ponte Nova												
<5 years	10·7	5·6	14·3	17·7	17·4	45·2	52·3	42·7	NA	NA	6·6	3·2, 9·9
5–9 years	12·7	4·7	9·8	13·1	14·1	28·4	34·9	24·1	NA	NA	3·4	1·0, 5·9
10–19 years	11·0	4·2	6·6	9·1	7·9	30·1	31·5	18·3	NA	NA	3·1	−0·1, 6. 2
20–59 years	6·5	3·4	5·6	8·5	8·7	10·1	10·7	10·0	NA	NA	0·9	0·3, 1·4
>60 years	0·1	0·0	0·3	5·9	2·8	8·1	13·8	8·1	NA	NA	1·7	0·7, 2·8
Pregnant women†	0·3	0·2	2·4	4·5	4·0	44·0	49·6	56·3	NA	NA	9·1	4·5, 13·7
Total coverage	7·3	3·4	6·0	9·2	8·8	16·9	19·1	14·6	17·3	17·7	1·7	0·9, 2·4
PBF contribution	74·9	92·2	97·8	68·9	80·7	54·0	59·5	68·4	56·6	47·0	−4·3	−7·1, 1·4
e-SUS AB contribution	NA	NA	NA	NA	NA	NA	NA	6·8	10·4	5·1	0·9	0·2, 1·6
Ubá											
<5 years	6·3	34·3	28·9	32·7	30·3	35·0	31·6	28·0	NA	NA	1·8	−1·5, 5·1
5–9 years	9·5	26·3	24·5	26·2	29·1	26·9	24·8	22·8	NA	NA	1·1	−1·1, 3·3
10–19 years	1·3	6·7	5·0	4·5	4·9	4·0	3·4	2·7	NA	NA	−0·1	−0·8, 0·6
20–59 years	0·2	3·5	2·4	3·1	3·3	3·4	2·5	1·9	NA	NA	0·1	−0·3, 0·6
>60 years	0·1	6·2	3·9	5·6	7·1	6·5	4·6	2·1	NA	NA	0·2	−0·8, 1·2
Pregnant women†	1·1	7·7	4·6	18·3	28·6	50·2	53·7	63·8	NA	NA	9·7	7·3, 12·1
Total coverage	1·6	8·5	6·7	7·6	8·1	8·2	6·9	5·8	5·11	10·3	0·3	−0·3, 0·9
PBF contribution	74·7	16·1	24·9	25·3	19·9	25·9	35·0	38·5	37·8	41·1	−0·3	−4·8, 4·2
e-SUS AB contribution	NA	NA	NA	NA	NA	NA	NA	0·6	8·6	1·8	0·5	−0·1, 1·1
Viçosa												
<5 years	38·2	24·9	43·3	46·7	43·9	36·3	44·3	46·4	NA	NA	1·5	−1·0, 4·1
5–9 years	23·7	16·6	23·0	25·9	36·7	40·8	25·4	23·6	NA	NA	1·3	−1·7, 4·2
10–19 years	9·5	6·6	8·7	10·2	25·8	33·5	13·00	13·8	NA	NA	1·8	−1·6, 5·2
20–59 years	5·3	5·9	6·8	8·0	9·4	9·4	9·2	10·8	NA	NA	0·8	0·6, 1·0
>60 years	0·1	0·0	0·4	1·1	6·7	6·7	10·0	17·7	NA	NA	2·4	1·3, 3·4
Pregnant women†	16·5	6·6	13·8	45·5	41·7	31·6	34·5	67·3	NA	NA	6·5	1·7, 11·3
Total coverage	9·4	7·6	10·1	11·7	16·1	16·9	13·2	15·5	16·08	17·42	1·0	0·5, 1·5
PBF contribution	70·7	88·1	72·8	61·0	40·6	41·9	54·0	44·9	38·4	30·2	−5·3	−7·8, 2·8
e-SUS AB contribution	NA	NA	NA	NA	NA	NA	NA	8·3	14·0	22·7	2·1	0·8, 3·4

NA, data not available; PBF, Programa Bolsa Família (Bolsa Family Programme); e-SUS AB, e-SUS Primary Care.

* Coefficient of linear regression.

† Specific estimate for pregnant women: number of live births from the previous year +10% (which referred to losses due to abortions and underreporting) and multiplied by the percentage of the female population of childbearing age.

In relation to total coverage, five municipalities showed a tendency to increase, whereas two (Juiz de Fora and Ubá) remained stable.

With regard to SISVAN web coverage as organised by life stage, in the majority of municipalities, the highest annual variation in coverage increase was concentrated in the group of pregnant women, except in Juiz de Fora, a municipality that did not present a significant increase in coverage in any monitored groups.

Children under 5 years of age comprised the next group with the greatest increase in coverage during the evaluation period, except in Ubá and Viçosa, where this increase was not significant.

In Manhuaçu, increased coverage in all groups was observed. In 2008, the lowest total coverage of SISVAN was in Ubá (1·6 %), and the highest was in Viçosa (9·4 %). In 2017, the lowest coverage was identified in Juiz de Fora (5 %), while the highest occurred in Manhuaçu (40 %). No municipality showed decreases in the number of users monitored. Adolescents and the elderly presented increased coverage in a smaller number of municipalities.

Between 2008 and 2017, the participation of the PBF in the monitoring of nutritional status showed a drop of 6·9 % in Muriaé and 6·4 % in Manhuaçu. In the other municipalities, participation remained stable.

**Fig. 1.**
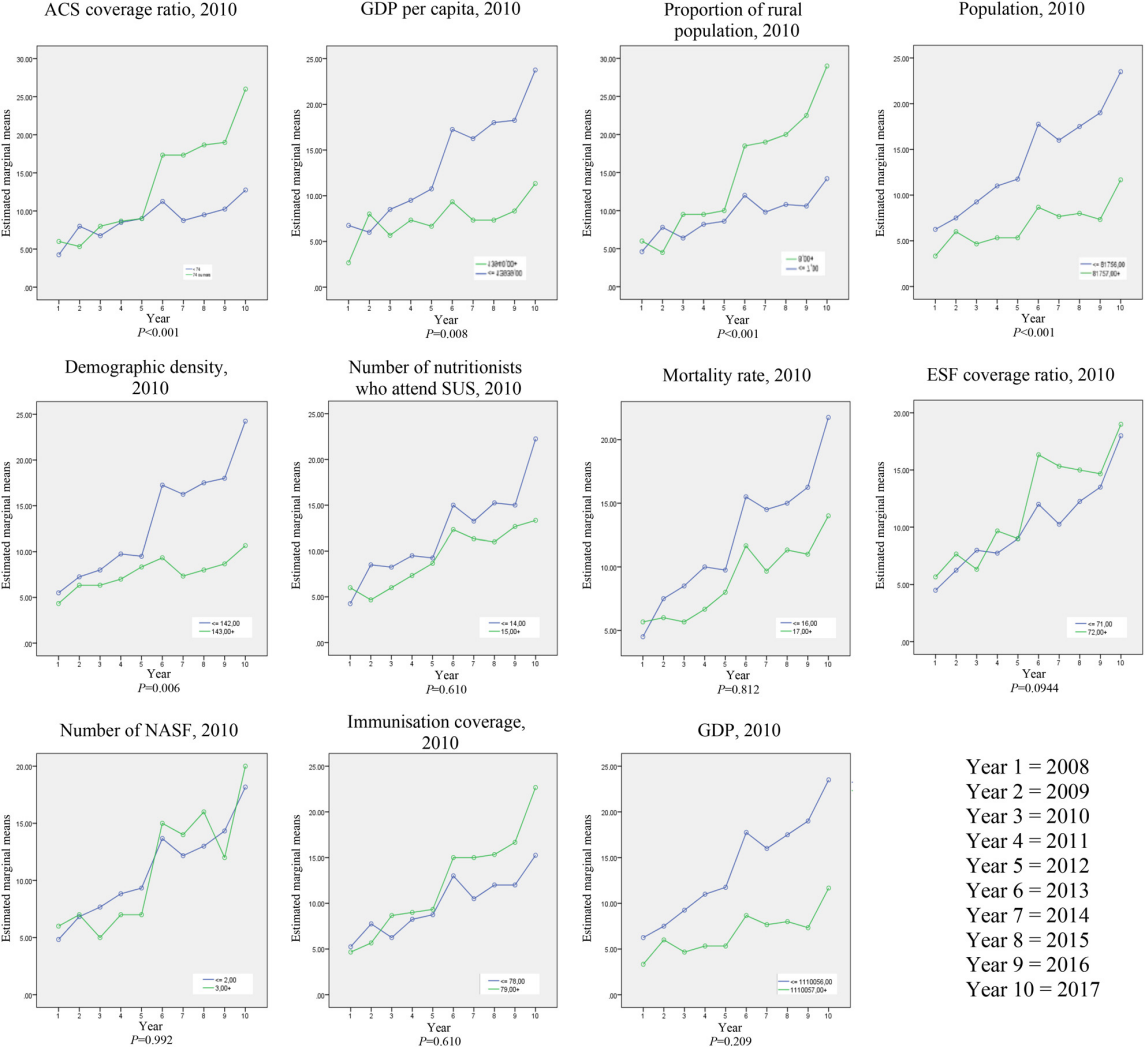
Association of nutritional status assessment coverage registered in the Sistema Nacional de Vigilância Alimentar e Nutricional (Food and Nutrition Surveillance System; SISVAN) web with the socio-economic, demographic and health variables in the municipalities evaluated. ACS, *agentes comunitários de saúde* (community health agents); GDP, gross domestic product; SUS, Sistema Único de Saúde (Unified Health System); ESF, Estratégia Saúde da Família (Family Health Strategy); NASF, Extended Core of Family Health.

Fig. 1 shows the association of nutritional status assessment coverage registered in the SISVAN web with the socio-economic, demographic and health variables in the municipalities evaluated. There was a positive association with proportion of rural population (*P* < 0·001) and coverage by ACS (*P* < 0·001) and a negative association with total population (*P* < 0·001), demographic density (*P* = 0·006) and GDP per capita (*P* = 0·008). Table 3 presents the evolution trend of the nutritional diagnosis of the population monitored in SISVAN in 2008.

**Table 3.** Nutritional diagnosis of users monitored by Food and Nutrition Surveillance System (Sistema Nacional de Vigilância Alimentar e Nutricional; SISVAN) in the health centre municipalities of Zona da Mata, Minas Gerais, 2008–2017*

	Year	Mean annual variation	95 % CI
	2008			2009	2010	2011	2012	2013	2014	2015	2016	2017
Malnutrition												
Preschool children	4·2	4·0	4·2	5·2	3·9	3·9	4·2	4·2	3·6	5·2	0·0	−0·2, 0·2
School-aged children	4·5	4·6	5·3	5·2	4·3	3·8	4·0	3·0	3·8	4·0	−0·2	−0·3, −0·0
Adolescents	6·9	4·3	5·3	4·2	4·2	4·2	3·8	3·4	4·3	3·5	−0·3	−0·4, −0·1
Adults	7·8	3·9	4·7	3·8	3·9	4·1	3·1	2·5	2·7	2·3	−0·4	−0·6, −0·3
Older adults	11·4	16·0	15·6	14·1	13·7	15·2	11·7	13·5	11·8	11·9	−0·3	−0·9, 0·4
Pregnant women	25·6	23·8	22·2	20·9	20·7	21·5	21·3	22·1	21·0	18·0	−0·5	−1·1, 0·0
Eutrophy												
Preschool children	87·2	87·1	86·4	84·3	85·7	84·1	83·7	85·0	86·2	87·3	−0·1	−0·5, 0·3
School-aged children	88·6	87·1	86·9	86·4	86·8	83·7	83·3	85·5	85·1	84·4	−0·4	−0·7, −0·1
Adolescents	74·0	74·9	72·2	72·3	71·4	67·7	66·9	64·5	67·8	66·6	−1·1	−1·4, −0·7
Adults	44·5	45·0	43·3	41·0	38·3	38·0	36·2	34·0	34·8	34·5	−1·4	−1·7, −1·0
Older adults	27·1	35·7	42·5	39·4	37·8	41·0	36·9	36·3	37·3	37·4	0·4	−0·5, 1·2
Pregnant women	36·0	41·9	38·9	40·7	37·6	40·6	37·8	37·9	36·0	37·6	−0·2	−1·1, 0·6
Overweight												
Preschool children	8·7	8·9	9·4	10·5	10·4	12·0	12·1	10·9	10·2	7·5	0·1	−0·2, 0·4
School-aged children	6·9	8·2	7·9	8·5	9·0	12·5	12·7	11·5	11·0	11·7	0·6	0·3, 0·9
Adolescents	19·0	20·8	22·5	23·5	24·4	28·1	29·3	32·1	28·0	29·9	1·3	1·0, 1·7
Adults	47·7	51·1	52·0	55·2	57·7	57·9	60·7	63·5	62·6	63·2	1·8	1·4, 2·2
Older adults	61·5	48·3	41·9	46·6	48·5	43·7	51·5	50·2	50·8	50·7	−0·1	−1·2, 1·0
Pregnant women	38·4	34·3	38·9	38·4	41·7	37·9	40·9	39·9	43·1	44·4	0·8	−0·1, 1·6

* Source: SISVAN.

## Discussion

Low SISVAN coverage was verified in all municipalities evaluated and supports findings from other studies^(12,13,16,17)^. Adolescents and the elderly showed lower coverage growth, which corroborates the results found in southern Brazil^(17)^. The Global Nutrition Report 2017 warned that more consistent data are needed for the adolescent age group to ensure good health at this critical stage of life, during which physical, mental and lifestyle changes occur. Many adolescents are affected by nutritional disorders but receive little food and nutritional surveillance^(10)^.

Elderly people are at risk of compromised nutritional status due to physical changes associated with ageing, as well as cognitive, psychological and social factors such as dementia, depression, isolation and limited income^(18)^. Malnutrition, which is common with ageing, negatively affects quality of life and increases health costs and the risk of short-term mortality^(19,20)^; therefore, evaluating the nutritional status of this group is critical.

A substantial increase in the follow-up of pregnant women was observed across all municipalities as a result of the greater attention given to prenatal and anthropometric data collection during prenatal care^(21)^. The reference value for this group was estimated based on the number of live births and the population of childbearing age in the year in question, since there was no reference value determined for this group^(15)^. As a result of this estimate, the proportion of pregnant women with nutritional status coverage superseded tha in 2014 and 2015.

The use of SISVAN as a system to manage the information on food and nutrition surveillance in primary care includes follow-ups registered in interfacing systems: the PBF and e-SUS AB. This is a strategy of the Department of Primary Care to restructure the information record of actions performed in primary care at the national level.

The participation of the PBF in obtaining data for Food and Nutrition Surveillance (Vigilância Alimentar e Nutricional; VAN) showed a downward trend in the period evaluated. VAN constitutes a conditionality of this income transfer programme; however, weaknesses in the joint intergovernmental and intersectoral coordination of the management of these health conditionalities were observed, which undermined the achievement of the goals^(22)^. Conversely, this downward trend in the participation of nutritional status assessment coverage is due to the reduction in the number of beneficiaries of the programme as a result of the crisis in Brazilian economic policy since the beginning of 2014^(23)^.

The cut in the nutritional monitoring of the beneficiary population of the programme in SISVAN showed the importance of this action in the available data of nutritional surveillance and reflected the positive result of this action as part of the health conditionalities of the income transfer programme. This result can be observed even in 2015, when 68·4 % of the nutritional data available in SISVAN came from the health conditionalities management system and expressed the nutritional status of the beneficiary population of the programme.

The participation of e-SUS AB, a strategy implemented in 2015, showed a slight but increasing trend in monitoring the nutritional status. In some Brazilian cities, the restructuring of SIS in primary care with e-SUS AB is already advanced. Although this restructuring represents a new strategy, benefits have already been shown in the work process of the professionals involved, mainly a reduction in the quantity of printed matter used in care records and procedures performed and in the duplication of work, as is the case of SISVAN. Although limitations exist with the integrated system, many benefits to Brazilian health services are expected^(24)^.

The positive association with the coverage by ACS verified in Zona da Mata-MG is based on the information generated for SISVAN and is produced in most municipalities by the ACS who collect the anthropometric data. Thus, this professional category plays a decisive role in the process of expanding coverage and monitoring SISVAN^(25)^.

In addition to the importance of ACS, financial issues can be highlighted. Higher values of GDP per capita were associated with lower coverage of nutritional status, which indicated that SISVAN coverage is linked more to the organisation of services rather than socio-economic conditions. The relationship between the wealth of countries measured by GDP or GDP per capita and the various health indicators is not linear^(26)^.

Municipalities with a proportion of rural population greater than 8 % had increased coverage of nutritional status compared with those with a smaller rural population, especially from 2013. Contrary to what is expected, access to the population is not a factor that makes covering the nutritional status a challenge.

The municipalities with lower demographic density tended to have increased coverage of nutritional status in SISVAN. Mendes^(27)^ stated that in defining the health care networks and the specific municipalities, regional characteristics, especially in areas of low population density, must be considered; the dispersion of the population can therefore favour more health services, facilitating the access of the resident population.

There was no association with the number of nutritionists from SUS. However, this variable showed a limitation because neither the workload nor the complexity of the work of these professionals was considered. This condition also occurred with the number of NASF-AB and the limitation was due to the presence of different professional categories in this core. In this study, the presence or not of nutritionists in NASF-AB was not investigated. The Extended Core of Family Health (NASF) established in 2008, today called the Expanded Core of Family Health and Primary Care (NASF-AB), was used as a strategic device for improving the quality of primary care. NASF-AB offers several initiatives employed to build, systematise, record and analyse or ‘how to do’ primary care. In the municipalities participating in our study, NASF-AB had recent implementation, but we want to present these data to verify the contribution of this multiprofessional team to the nutritional care in AB, considering the purposes of this centre.

Finally, no significant difference in the coverage of nutritional status was found when there was an increase in GDP; this finding indicated that the incorporation of VAN practices into daily health services did not depend exclusively on the availability of financial resources, but also other aspects such as better organisation of nutritional care and better structuring of primary care services. The political commitment to public health and SUS by managers and professionals responsible for food and nutrition actions in the territories is also a relevant aspect in this reorganisation^(15)^, and should include policies aimed at permanent education and the valourisation of professionals with decision-making roles in the territories.

The monitored population seeking health services probably exhibits a higher occurrence of health problems, including malnutrition and obesity, than does the general population. This factor may lead to an overestimation of nutritional disorders. In this study, because these actions were started recently, there were insufficient data on an assessment of the trend of coverage of the evaluation of food consumption markers. The data are still inconsistent and interrupted. However, considering the importance of the food aspect in the VAN, this evaluation should engender future studies.

When the results are interpreted, limitations that can lead to the possibility of bias from the use of secondary data, such as underreporting or errors in the process of generating the information, should be considered.

### Implications for public health

This study revealed the low coverage of the nutritional status of certain populations and demonstrated the need to obtain pertinent and reliable data to allow routine monitoring of nutrition and health; this action will facilitate the detection of trends that help in making decisions and formulating health actions and policies in a particular country or region. A food and nutrition surveillance system should include a broad set of appropriate and quality indicators that are collected at a satisfactory frequency and that focus on groups vulnerable to food and nutrition insecurity. Knowing the factors that influence the trend of nutritional monitoring in health services enables decision makers to identify emergencies, develop effective strategies before the onset of a crisis, and facilitate the timely initiation of response and risk reduction efforts.
